# A systematic review and meta-analysis of Vitamin D status and clinical outcomes in critically ill neonates

**DOI:** 10.3389/fnut.2026.1770536

**Published:** 2026-06-15

**Authors:** Nowel Tan, Charmaine Shi Mei Lee, Eirena Beh, Darolyn Jia Lin Tan, Ruther Teo Zheng, Daniel Chan

**Affiliations:** 1 Department of Paediatrics, KK Women’s and Children’s Hospital, Singapore, Singapore; 2 Yong Loo Lin School of Medicine, National University of Singapore, Singapore, Singapore; 3 Endocrinology Service, Department of Paediatrics, KK Women’s and Children’s Hospital, Singapore, Singapore; 4 Duke-NUS Medical School, Singapore, Singapore

**Keywords:** cholecalciferol, clinical outcomes, critically ill, neonates, NICU, Vitamin D

## Abstract

**Introduction:**

Vitamin D is key in immunoregulation and impacts critical care outcomes. With a high prevalence of neonatal Vitamin D deficiency (VDD), we aim to evaluate its association with clinical outcomes in the neonatal intensive care unit (NICU).

**Methods:**

Systematic search of PubMed, EMBASE, Web of Science and Scopus was conducted. Primary outcomes included mortality and incidence of sepsis, while secondary outcomes were length of stay (LOS), incidence of bronchopulmonary dysplasia (BPD), and need for mechanical ventilation (MV). Eligible studies included preterm and term NICU neonates with 25-hydroxyvitamin D levels reported on admission. Definition of VDD was based on individual studies due to significant inter-study heterogeneity, however further sub-analyses with VDD definitions of ≤ 30 ng/mL and Vitamin D sufficient (VDS) > 30 ng/mL were performed to evaluate possible dose–response relationships. Pooled estimates were calculated with random-effects model due to study heterogeneity.

**Results:**

Among 2,735 articles, 18 studies met predefined inclusion criteria (*n* = 1981 patients). VDD was associated with increased incidence of sepsis [odds ratio (OR) 2.31, 95%CI 1.50–3.57], length of hospital stay (mean difference 4.52 days, 95% CI 1.66–7.37) and need for MV (OR 1.67, 95%CI 1.09–2.55), in comparison to VDS neonates. Further subgroup analyses for preterm (OR 2.05, 95%CI 1.04–4.04) and term neonates (OR 3.96, 95%CI 1.68–9.35) continued to show significant association between VDD and sepsis. No differences were observed between both groups for mortality and incidence of BPD. Further subgroup analyses based on Vitamin D levels found that those ≤ 30 ng/mL had no significant difference (mean difference 0 days, 95%CI -1.89 - 1.89) in terms of length of hospital stay.

**Conclusion:**

VDD in critically ill neonates is significantly associated with increased odds of sepsis, need for MV, and longer LOS. However, these findings are limited by significant heterogeneity across studies. This warrants further interventional studies, involving both term and preterm neonates, to examine the potential of optimization of Vitamin D levels to improve critical care outcomes in NICU.

## 1 Introduction

Vitamin D is an essential substrate with an active role in calcium homeostasis and metabolic bone health (1). It is a fat-soluble compound synthesized endogenously in the skin via conversion from 7-hydrocholesterol by ultraviolet (UV) B light, and/or obtained via dietary sources such as fatty fish, cod-liver oil, eggs, fortified products (milk, yoghurt, breakfast grains) or supplements (2, 3). Over the years, more studies have revealed the extensive role of Vitamin D in immunoregulation, endothelial function and anti-microbial activity (4). Due to its multi-systemic involvement that is crucial in times of critical illness, there are several hypotheses on the association between Vitamin D deficiency (VDD) and clinical outcomes in the critically ill population, with considerations for Vitamin D normalization (5) in order to improve outcomes in this population.

VDD has been acknowledged as a global health problem, with an estimated one billion people being affected worldwide (6). In particular, there is greater awareness of the higher prevalence of VDD in neonates, which is concerning given its association with neonatal morbidities (7). A study by Park et al. (8) on a population of preterm infants in South Korea found that 98.9% had vitamin D insufficiency and half of them were severely deficient. Another similar study in Thailand by Ariyawatkul et al. (9) that included both term and preterm neonates also found high prevalence of VDD. This phenomenon has been reported in critically ill neonates as well - a study in Tanzania showed that around 80% of neonates in a tertiary care hospital’s NICU had vitamin D insufficiency (10). Postulated aetiologies of vitamin D deficiency in neonates include inadequate nutrition, maternal vitamin D deficiency with exclusive breastfeeding, understandably low UV exposure, and use of drugs such as corticosteroids (11). Consequently, many studies go on to report associations between VDD and neonatal critical care outcomes (12) including bronchopulmonary dysplasia (BPD), neonatal sepsis and necrotizing enterocolitis in both term and preterm neonates. The association between VDD and neonatal sepsis may be explained by the immunoregulatory role of vitamin D; adequate vitamin D status supports antimicrobial peptide production and lymphocyte activity, while deficiency may impair these host defense mechanisms (13). Similarly, in a murine model, vitamin D limits intestinal damage caused by necrotizing enterocolitis by downregulating expression of Toll-like receptor 4 (14).

Across adult and pediatric critical care populations, there has also been greater recognition of the association of VDD with severe illnesses, critical care outcomes and mortality. Multiple studies involving adult intensive care units found association of VDD with survival duration (15) and clinical outcomes. Similarly, meta-analyses conducted on critically ill pediatric populations reported association of VDD with disease severity and mortality (16). Hence, efforts have made way for large randomized controlled trials (RCTs) in these populations, with the VITdAL-ICU trial investigating the effect of Vitamin D supplementation on critically ill adult patients (17) and similarly-designed multicentered RCTs in pediatric intensive care units (18) (VITdALIZE-KIDS) evaluating the effect of rapid normalization of Vitamin D levels on critical care outcomes. Yet, the impact of VDD on the neonatal critical care population remains understudied. In NICU, the prevalence of VDD critically ill neonates has been found to be significantly high (19). There is a dearth of data on how Vitamin D status affects critical care outcomes in NICU patients. Hence, our team aims to perform a systematic review and meta-analysis to investigate the association of VDD with essential clinical outcomes in critically ill preterm and term neonates. Our long-term objective is to gain insights on the potential benefits of Vitamin D normalization in critically ill neonates, eventually hoping to pave the way for further robust interventional studies examining the role of Vitamin D in influencing clinical outcomes in NICU babies.

## 2 Methods

### 2.1 Search strategy and eligibility criteria

Our study protocol was registered in PROSPERO (CRD420251132667). We conducted a systematic review across four databases (PubMed, EMBASE, Web of Science and Scopus) for articles published from inception until 23rd August 2025. A comprehensive search strategy (Supplementary material 1) was developed together with a university librarian. No language restrictions or specific filters were applied. Our eligibility and exclusion criteria are as described below.

#### 2.1.1 Inclusion criteria

- Population: Neonatal population (including preterm and term infant patients) admitted to NICU
- Exposure: Measured and reported on Vitamin D status on Vitamin D status on admission, in particular 25-hydroxyvitamin D levels
- Outcome: Reported on outcomes of interest: mortality, hospital length of stay, incidence of sepsis, bronchopulmonary dysplasia and need for mechanical ventilation
- Types of study: Randomized controlled trials, observational studies (prospective and retrospective), case–controlled and cross-sectional studies.

#### 2.1.2 Exclusion criteria

- Any studies involving neonates not admitted to NICU, but admitted to general ward or high dependency.
- Any studies involving adults or pediatrics populations
- Case reports, reviews, and abstracts
- Studies which did not examine associations between Vitamin D levels and outcomes of interest

### 2.2 Data extraction

Systematic screening of articles was performed with the assistance of the Covidence web (20). After removal of duplicates, title and abstract screening was performed by four authors (NT, EB, CL and DT), followed by full text reviews. Any disagreements between reviewers were resolved through discussion and adjudication by an independent fifth reviewer (DC). The data extracted included: (1) study characteristics (year of publication, type of study, country and continent of study), (2) baseline maternal (age, ethnicity, antenatal factors such as maternal Vitamin D levels and antenatal steroids use) and patient demographics (gestational age, sex, anthropometry details, APGAR scoring), (3) interventions (if applicable) and (4) outcome measures. Patients must also have had their 25-hydroxyvitamin D levels measured at baseline to allow for further categorization into VDD and Vitamin D sufficient (VDS) groups. According to global consensus and recommendation, VDD has been defined as <20ng/mL and VDS as ≥ 20 (57). However in view of significant heterogeneity of VDD and VDS definitions across papers, the definition of VDD and VDS groups were based on levels as defined by the individual studies. Further subgroup analyses were performed to compare between preterm and term babies, as well as across different VDD levels (i.e., VDD ≤ 30 ng/mL and VDS > 30 ng/mL) to ensure a robust examination of the relationship between Vitamin D levels and critical care outcomes.

Our primary outcomes defined a priori as all-cause mortality, which included mortality across timeframes from in-hospital mortality to 28-, 60- and 90-day mortality, as well as the incidence of sepsis. The time of diagnosis of sepsis was either at the point of or soon after NICU admission. In terms of definitions of sepsis, they varied across papers but generally included late-onset sepsis, culture-positive sepsis, biochemical markers such as C-reactive protein, and scoring systems like the neonatal Sequential Organ Failure Assessment (nSOFA). Secondary outcomes assessed were length of hospital stay, need for mechanical ventilation, and incidence of BPD. The definition of BPD was generally based on Jobe and Bancalari (21); defined as a chronic lung condition in neonates requiring prolonged oxygen therapy (≥ 28 days) and/or mechanical ventilation at 36 weeks corrected age, with severity classified based on differing oxygen requirements. Meanwhile, length of stay specifically refers to in-hospital stay, instead of duration of intensive care unit stay.

### 2.3 Quality assessment

For cohort and case–control studies, the risk of bias was assessed using the Newcastle–Ottawa Quality Assessment Scale (22). It utilizes a nine-star rating system to assess quality of non-randomized studies over three main domains of study sample selection, comparability, and outcome/exposure (Supplementary material 2). For cross-sectional studies, the risk of bias was assessed using a validated adaptation (23) of the Newcastle–Ottawa Quality Assessment Scale (NOS-xs) that similarly involves three main domains of (i) study sample selection, (ii) assessment of exposure(s) and outcome(s), and (iii) confounding factors.

Meanwhile, RCTs (Supplementary material 3) were assessed using the Grading of Recommendations Assessment, Development, and Evaluation (GRADE) Risk of Bias version 2 (GRADE RoB2) tool (24). The tool provides a systematic framework for grading quality of evidence based on its randomization process, outcomes as well as any deviations, missing data or bias in selection of reported results. Subsequently, an overall risk of bias grade will be assigned.

### 2.4 Statistical analysis

Statistical analyses were performed using R version 4.5.1 (R Core Team, 2025). The random effects model was used to calculate pooled estimates and 95% CI. Dichotomous outcomes were reported as odds ratio (OR) with 95% CI, while continuous outcomes were reported as mean difference (MD) with 95% CI. Inter-study heterogeneity was assessed using I^2^ statistic. Egger’s test was used to assess for presence of publication bias according to our funnel plots.

## 3 Results

### 3.1 Study selection

**Figure 1:**
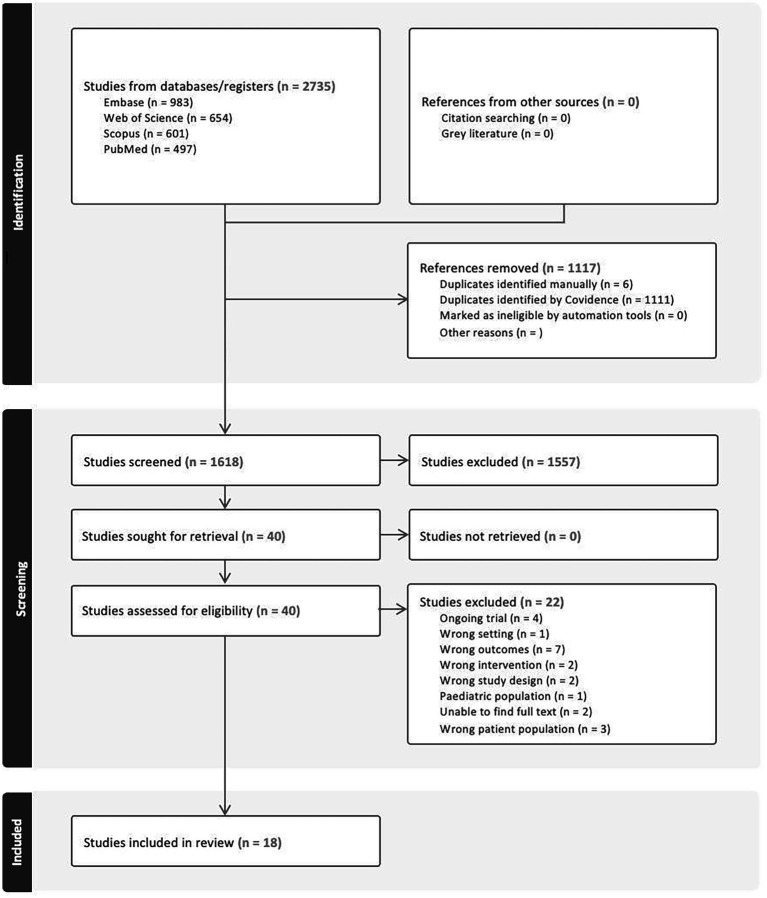
According to preferred reporting items for systematic reviews and meta-analyses (PRISMA) guidelines.

The study reported according to the Preferred Reporting Items for Systematic Reviews and Meta-Analyses (PRISMA) guidelines (25) (Figure 1). Our search yielded a total of 2,735 articles, of which 1,117 were duplicates and excluded. Eventually, 18 studies fulfilled inclusion criteria for the review. In total, we included 4 case–control studies (26–29), 2 RCTs (30, 31), 11 cohort studies (32–42), and 1 cross-sectional study (19). Regarding risk of bias evaluation, our observational studies were rated well and both of our RCTs were rated as ‘some concern’ and ‘low’ risk of bias, respectively.

### 3.2 Study characteristics

Baseline study characteristics and demographics of neonates and their mothers are shown in Table 1. A total of 1981 critically ill preterm and term neonates admitted to NICU were evaluated. Across studies, the sex distribution was well-balanced (965 males, 51.9% - excluding 2 studies where this information was not available). In terms of gestational age, majority (27, 30–32, 34, 35, 38–41) of the studies (*n* = 10) reported on preterm babies, while 3 studies (26, 29, 36) focused on term babies. As for the remaining studies, 3 of them (33, 37, 42) identified patients based on birth weight, 1 (28) reported on late preterm neonates (gestational age ≥ 35 + 0 weeks) and the other (19) included all neonates admitted to NICU regardless of gestational age. Eight studies (19, 26, 30, 31, 34, 38, 40, 41) were conducted in upper-middle-income countries, while remaining studies took place in high (32, 33, 35, 37, 42) and low-middle-income countries (27–29, 39), as defined by the World Bank’s classification (43) - 1 study did not provide relevant information. Amongst the nine studies (30–32, 34, 35, 37, 39, 41, 42) that reported on use of antenatal steroids, it varied significantly amongst studies likely due to differing clinical practices and accessibility to medications.

**Table 1:** Study characteristics of included studies.

Study	Continent	Country	Country income	Type of study	Patient population^*^		Gender (n, % male)	BW in grams (mea*n*, SD)	Mother’s vit D in ng/mL (median, IQR)	Proportion of mother’s antenatal steroid use (n, %)
					Gestational age (weeks)	BW (grams)				
Karatekin et al. (26)	Mixed	Turkey	Upper-middle	Case–control	39 + 0–40 + 6	-	-			
Onwuneme et al. (32)	Europe	Ireland	High	Cohort	< 32 + 0	-	40 (44)	1193 (275)	24.2 (14.1–38.0)	86 (95)
Cetinkaya et al. (41)	Mixed	Turkey	Upper-middle	Cohort	≤ 36 + 0	-	75 (51.7)	-	-	75 (54.5)
Puthuraya et al. (33)	North America	United States of America	High	Cohort	-	Very low birth weight ≤ 1,250 g	47 (52.8)	-	21 (4–44)	-
Prasad et al. (27)	Asia	India	Low-middle	Case–control	34 + 0 to 36 + 6	-	75 (62.7)	-	-	-
Dhandai et al. (28)	Asia	India	Low-middle	Case–control	≥ 35 + 0	-	77 (18.3)	-	-	-
Kim et al. (42)	Asia	Korea	High income	Cohort	-	Very low birth weight < 1,500 g	99 (52.7)	1104.7 (298.1)	-	97 (51.6)
Dogan et al. (34)	Mixed	Turkey	Upper-middle	Cohort	≤ 32 + 0	-	36 (50)	-	-	30 (41.7)
Al-Matary et al. (35)	Asia	Saudi Arabia	High	Cohort	≤ 34 + 0	-	97 (55.7)	1,390 (410)	-	111 (63.8)
Mahmoud and Elela (36)	Mixed	Egypt	Low-middle	Cohort	≥ 37 + 0	-	83 (56.1)	-	-	-
Mosayebi et al. (19)	Asia	Iran	Upper-middle	Cross-section	All neonates admitted to NICU	All neonates admitted to NICU	40 (40)	-	-	-
Matejek et al. (37)	Europe	Czech Republic	High	Cohort	-	Birth weight <1,500 g	48 (59.3)	1,190 (930–1,340)	38 (24–66)	79 (97)
Jafari et al. (38)	Asia	Iran	Upper-middle	Cohort	< 37 + 0	-	26 (23)	-	-	-
Ge et al. (30)	Asia	China	Upper-middle	RCT	< 32 + 0	-	57 (46)	-	-	20 (16.1)
Saggi (39)	-	-	-	Cohort	< 32 + 0	-	67 (67)	-	-	43 (43)
Boskabadi et al. (31)	Asia	Iran	Upper-middle	RCT	< 34 + 0	-	56 (56)	-	-	86 (86)
Mohamed et al. (29)	Mixed	Egypt	Low-middle	Case–control	≥ 37 + 0	-	42 (46.7)	-	-	-
Cetin et al. (40)	Mixed	Turkey	Upper-middle	Cohort	34 + 0 to 36 + 6	-	-	-	-	-

Data are presented as numbers and frequency for categorical variables, and mean (standard deviation) or median (interquartile range) for continuous variables.

^*^Patient population as defined by individual study, including gestational age (weeks) and birth weight (grams).

SD, standard deviation; *n*, number; IQR, interquartile range.

### 3.3 Primary outcomes

#### 3.3.1 All-cause mortality

**Figure 2:**
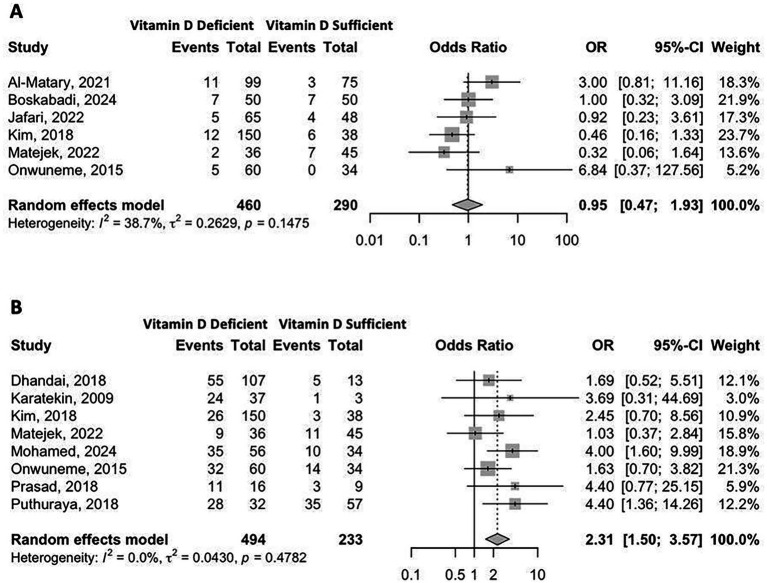
Forest plots showing the effect of vitamin D deficiency on primary outcomes **(A)** All-cause mortality in critically ill VDD neonates as compared to VDS neonates, **(B)** Incidence of sepsis in critically ill VDD neonates compared to VDS neonates.

Six studies (31, 32, 35, 37, 38, 42), comprising 750 of the 1981 included neonates, provided extractable data on all-cause mortality and were included in the mortality-specific meta-analysis. Among these studies, VDD was not significantly associated with increased odds of all-cause mortality compared with VDS (pooled OR 0.95, 95% CI 0.47–1.93) (Figure 2A). There is moderate heterogeneity across these studies. (I^2^ = 38.7%, *p* = 0.1475).

#### 3.3.2 Incidence of sepsis

Eight studies (26–29, 32, 33, 37, 42) that encompassed 727 patients reported on the incidence of sepsis. Critically ill VDD neonates had significantly increased odds of sepsis compared to that of VDS neonates (pooled OR 2.31, 95%CI 1.50–3.57) (Figure 2B). There was low heterogeneity across these studies. (I^2^ = 0.0%, *p* = 0.4782).

**Figure 3:**
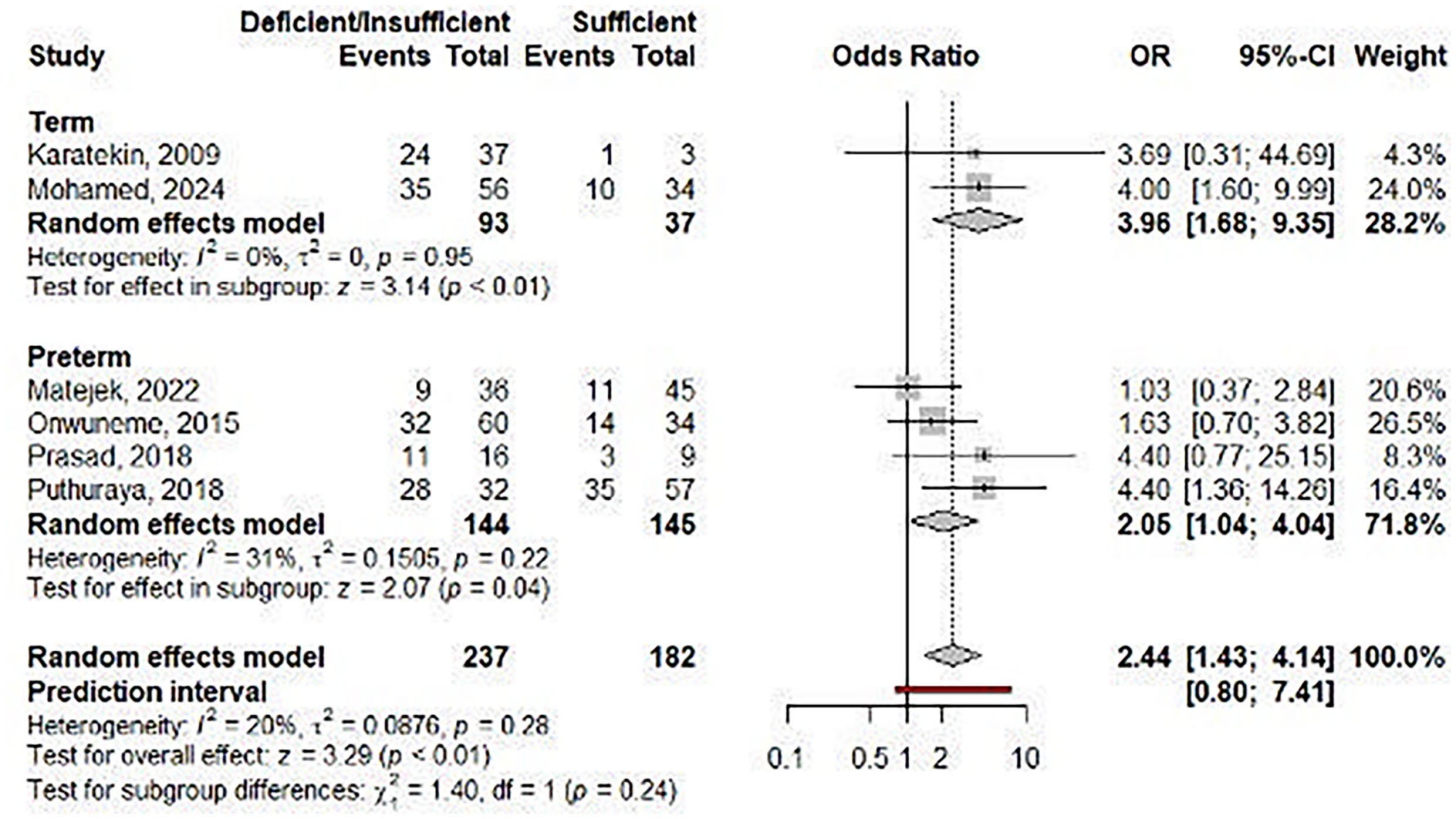
Subgroup analysis of association of VDD and incidence of sepsis for term and preterm neonates, respectively.

Among these studies, two studies (26, 29) focused on full-term babies (≥ 37 weeks) and further sub-analysis focusing on full-term babies found that VDD in critically ill term babies was significantly associated with increased odds of sepsis (pooled OR 3.96, 95%CI 1.68–9.35) compared to the VDS group (Figure 3). There is low heterogeneity across these studies. (I^2^ = 0.0%, *p* = 0.95).

On the other hand, four studies (27, 32, 33, 37) reported mainly on premature babies, for which the odds of sepsis was higher in the critically ill VDD neonates than VDS neonates (pooled OR 2.05, 95%CI 1.04–4.04) (Figure 3). Across these studies, there is moderate heterogeneity (I^2^ = 31%, *p* = 0.22).

#### 3.3.3 Publication bias

Publication bias for the primary outcomes are presented as funnel plots. Overall, there was no publication bias in the papers reporting on mortality and incidence of sepsis. Egger’s test yielded a *p*-value of 0.217 for studies on mortality (Supplementary material 4A) and 0.434 for that on incidence of sepsis (Supplementary material 4B), indicating little publication bias for both primary outcomes.

### 3.4 Secondary outcomes

#### 3.4.1 Length of stay

**Figure 4:**
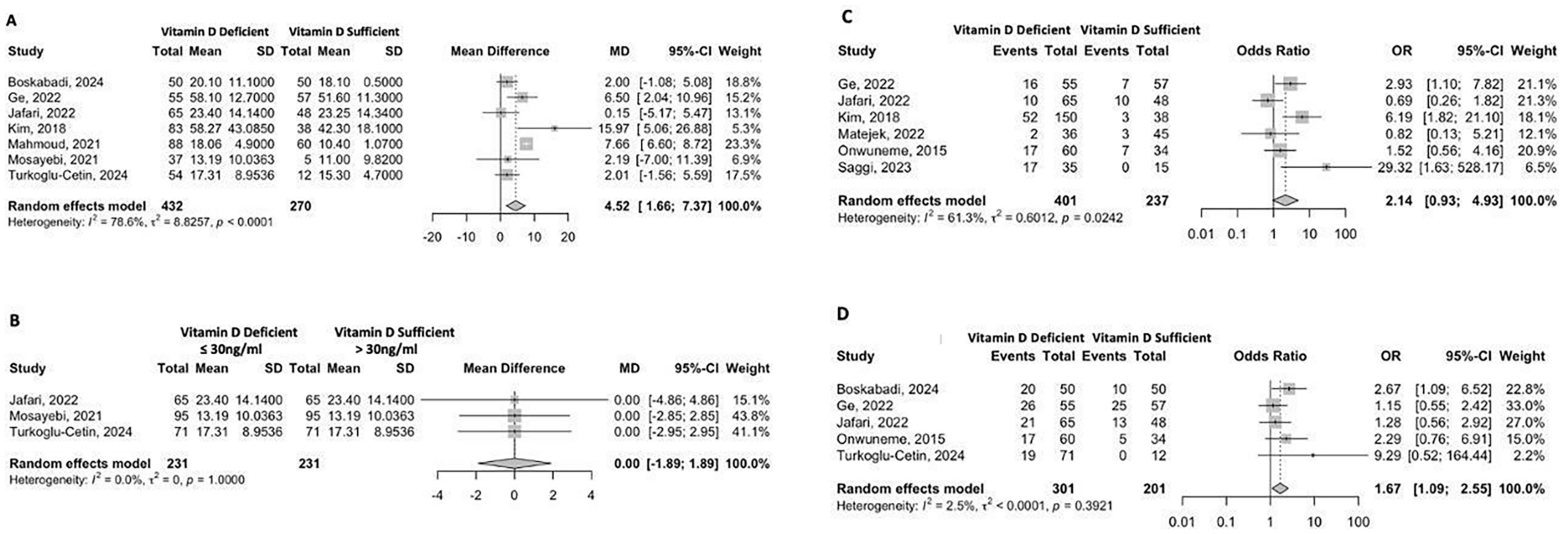
Forest plots showing the effect of VDD on secondary outcomes and subgroup analyses: **(A)** Length of hospital stay in critically ill VDD neonates compared to VDS, **(B)** subgroup analyses comparing length of hospital stay between studies defining VDD as ≤ 30 ng/mL and VDS as > 30 ng/mL, **(C)** incidence of BPD in critically ill VDD neonates in comparison to VDS neonates, **(D)** need for mechanical ventilation in critically ill VDD neonates in comparison to VDS neonates.

Seven studies (19, 30, 31, 36, 38, 40, 42) reported on the length of stay in hospital (Figure 4A). There was a longer length of hospital stay in critically ill VDD neonates compared to that of VDS neonates (mean difference 4.52 days, 95%CI 1.66–7.37). There is significant heterogeneity across studies (I^2^ = 78.6%, *p* = <0.001).

In a subgroup analysis (Figure 4B) of three studies (19, 38, 40), neonates with 25-hydroxyvitamin D levels ≤ 30 ng/mL were compared to those with levels >30 ng/mL. There was no significant difference (mean difference 0 days, 95%CI -1.89 - 1.89) in terms of length of hospital stay for both groups.

#### 3.4.2 Incidence of BPD

There were six studies (30, 32, 37–39, 42) that reviewed incidence of BPD (Figure 4C) VDD was not significantly associated with increased odds of BPD compared to neonates with VDS (pooled OR 2.14, 95%CI 0.93–4.93). There is substantial heterogeneity across studies (I^2^ = 61.3%, *p* = 0.024).

#### 3.4.3 Need for mechanical ventilation

Five studies (30–32, 38, 40) reported on the need for mechanical ventilation (Figure 4D). The odds of needing mechanical ventilation was higher amongst the critically ill VDD neonates as compared to the VDS group (pooled OR 1.67, 95%CI 1.09–2.55). There is minimal heterogeneity across studies (I^2^ = 2.5%, *p* = 0.3921).

## 4 Discussion

Key findings from our study suggest that VDD is associated with increased incidence of sepsis, longer duration of hospital stay, and increased need for mechanical ventilation in critically ill neonates. Further subgroup analysis comparing critically ill preterm and term neonates also noted increased odds of sepsis in both groups, respectively. However, there was no association with increased mortality and incidence of BPD.

Our findings align with other studies that focus on the association between VDD and risk of sepsis in the neonatal population. A systematic review by Bitew et al. (13) similarly found that neonates with low vitamin D levels were at increased risk for neonatal sepsis. Yang et al. (44). also reported the association of development of early-onset sepsis in term neonates with VDD. However, it is crucial to note that these studies mostly focus on either the neonatal population in general or in term or preterm infants separately, instead of critically ill neonates as a study population in particular. Regarding incidence of BPD, another meta-analysis by Yang et al. (45). found that there was no significant difference between Vitamin D supplementation and control groups, which is also in keeping with our findings of lack of association between VDD and BPD incidence in the NICU neonates.

Comparatively, the association between VDD and critically ill adult and paediatric populations are more widely studied. A recent meta-analysis (16) on critically ill pediatric patients found that VDD was associated with increased mortality and need for inotropic support. Meanwhile, in the critically ill adult population, Kim et al. (46) also suggests that VDD increases susceptibility for severe infections and mortality. VITdAL-ICU (17), a randomized clinical trial looking into the effect of high-dose Vitamin D3 on hospital length of stay in critically ill adult patients, observed lower hospital mortality in the severe VDD subgroup. Similarly there have also been multiple studies examining the association between VDD and clinical outcomes in critically ill children admitted to the PICU. VITdAL-PICU (47) a phase 2 study, examined the efficacy and safety of Vitamin D loading doses to rapidly restore Vitamin D levels in critically ill paediatric patients (>37 weeks corrected gestational age to <18 years old). This has shown encouraging results and was followed by the ongoing phase 3 multi-centre, double blind randomized controlled trial, the VITdALIZE-KIDS (18), aiming to evaluating whether rapid normalization of Vitamin D levels could potentially improve critical care outcomes in the paediatric population. However, to the best of our knowledge, there have been no RCTs designed specifically for critically ill neonates. Thus, our study aims to directly fill this clinical gap, looking into the association between VDD and important neonatal critical care outcomes, highlighting the unmet need to design robust interventional studies in this vulnerable population.

Exact mechanistic pathways between Vitamin D deficiency and incidence of sepsis remains undelineated, but plausible explanations may lie within the fact that Vitamin D is a key immunoregulator (48), aside from its well-known effect on calcium homeostasis. Activated Vitamin D mediates production of antimicrobial peptides such as cathelicidin (4), which result in activation of Toll-like receptors (TLR) that then stimulates macrophages. Immune cells such as B cells and T cells (49) also express Vitamin D receptor (VDR) allowing for immunoregulation. There are also implications on endothelial function and hemodynamics as Vitamin D affects vascular permeability (50) by acting as a transcriptional regulator in nitric oxide synthesis. Overall, these processes contribute to the pathogenesis of sepsis via immunoregulatory pathways and its active roles in the innate and adaptive immune system.

As for respiratory implications, there is increasing evidence on the association between VDD and pulmonary disease in very preterm infants (51). Lung maturation via stimulation of surfactant protein and modulation of alveolar cells require activated Vitamin D as a key factor (52). Taylor et al. (53) reported that inhalational Vitamin D may be a novel way in treatment of respiratory morbidities via increasing levels of alveolar surfactant protein B. While our study reported significant association between VDD and need for mechanical ventilation in critically ill neonates, it is notably not significantly associated with incidence of BPD. Contributory factors for this finding may be due to the heterogenous definitions of BPD across studies and varied institutional practices.

In light of our findings of association of VDD with multiple outcomes in critically ill neonates and potential mechanisms as reviewed above, strategies into optimization of Vitamin D levels in this group of patients should be investigated further. Natalia et al. (54) reported an ancillary study of a masked RCT involving administration of enteral Vitamin D at 200 IU/day and 800 IU/day in critically ill extremely preterm infants and found that 800 IU/day dosing safely corrected VDD by postnatal day 14. Amongst the paediatric population with sepsis, Wang et al. (55) performed an RCT involving administration of single dose of 150,000 IU of cholecalciferol and found lower incidence of septic shock in the treatment group. Similarly, the VITdAL-PICU RCT (47) involving critically ill children also found a single 10,000 IU/body weight (kg) dose can safely normalize Vitamin D levels. Overall, there are differing methodologies in the optimization of Vitamin D levels including a single loading dose as compared to multiple lower doses, as well as various modes of administration that warrants more in-depth interventional studies.

The strength of our study lies within our rigorous and comprehensive search strategy, unrestricted by time period. Also, we have utilized a stringent inclusion criterion to ensure that the focus is on critically ill neonates specifically but broad enough to include both preterm and term neonates, providing a holistic report on the impact of VDD in NICU. Additionally, the included studies are of high quality, reducing the risk of inherent methodological biases and ensuring fair comparability between variables. For our primary outcomes, there was also no significant publication bias reported that could cause potential skewing of results and hence inaccurate evaluation of VDD’s effect size on mortality and incidence of sepsis. Further subgroup analyses stratifying preterm and term neonates provide additional insights on the association of VDD for different gestational ages.

However, there are several limitations within our study. One of the key limitations is that of significant heterogeneity in the definitions of VDD across individual studies, which hinders direct comparison between Vitamin D levels and clinical outcomes. Hence, this could impact external validity, and findings should be interpreted cautiously. We attempted to overcome this by performing subgroup analyses based on specific VDD definitions, finding that VDD ≤ 30 ng/mL was not significantly associated with LOS. Secondly, as our search yielded mostly preterm neonates, this could contribute to critically ill term neonates being under-represented and hence affect the generalizability of our results to the NICU population. Additionally, multiple studies did not provide information regarding birth weight, which is a critical determinant of neonatal mortality and morbidity (56), and this could potentially confound and impact neonatal clinical outcomes. This could possibly be attributed to some studies opting to use gestational age as a surrogate marker or limited by access to this information, which is not ideal and should be taken into consideration. Meanwhile, the relatively small number of eligible studies could have impacted our overall study population’s sample size and limit overall generalizability of our findings. Finally, most of the studies included were observational in nature, hence limiting the ability to infer causative relationships and this should be considered in the interpretation of our findings.

## 6 Conclusion

In conclusion, our study demonstrated associations of VDD with increased incidence of sepsis, need for mechanical ventilation and longer duration of hospital stay in critically ill neonatal patients. However, large-scale prospective studies are required to further evaluate this association, exploring possible dose–response relationships with differing severities and cutoffs of Vitamin D levels. Additionally, better inclusivity of such studies to involve both term and preterm neonates would allow for greater generalizability of findings and hence application into clinical practice. Finally, there is also potential to explore interventional studies with well-designed RCTs to investigate the clinical benefits of normalizing Vitamin D in critically ill neonates.

## Data Availability

The raw data supporting the conclusions of this article will be made available by the authors, without undue reservation.
