# Supplementary material for: A systematic review and meta-analysis of Vitamin D status and clinical outcomes in critically ill neonates
Source: Front Nutr. 2026 Jun 15;13:1770536. doi: 10.3389/fnut.2026.1770536 (PMC13312809; doi:10.3389/fnut.2026.1770536)
Supplement: Supplementary file 1 [file Data_Sheet_1.pdf]

Supplementary 1: Search strategy

Database	Search strings
Pubmed (Medline)	(("Vitamin D"[Mesh]) OR (Vitamin D[Title/Abstract] OR Cholecalciferol[Title/Abstract] OR Hydroxycholecalciferols[Title/Abstract] OR Calcifediol[Title/Abstract] OR Calcitriol[Title/Abstract] OR Dihydroxycholecalciferols[Title/Abstract] OR 25-Hydroxyvitamin D 2[Title/Abstract] OR Ergocalciferols[Title/Abstract])) AND<br><br>(("Intensive Care Units"[Mesh] OR "Critical Care"[Mesh]) OR (Intensive Care Units[Title/Abstract] OR Intensive Care Units, Pediatric[Title/Abstract] OR Intensive Care Units, Neonatal[Title/Abstract] OR Critical Care[Title/Abstract] OR Critical Illness[Title/Abstract]))
Embase	'vitamin d'/exp OR '25 hydroxyvitamin d'/exp OR 'colecalciferol derivative'/exp OR 'calcifediol'/exp OR 'ergocalciferol derivative'/exp OR 'vitamin d':ab,ti OR '25 hydroxyvitamin d':ab,ti OR 'colecalciferol derivative':ab,ti OR 'calcifediol':ab,ti OR 'ergocalciferol derivative':ab,ti AND<br><br>('newborn intensive care'/exp OR 'pediatric intensive care unit'/exp OR 'neonatal intensive care unit'/exp OR 'critically ill'/exp OR 'newborn intensive care':ab,ti OR 'pediatric intensive care unit':ab,ti OR 'neonatal intensive care unit':ab,ti OR 'critically ill':ab,ti)
SCOPUS	(TITLE-ABS-KEY ("Vitamin D" OR cholecalciferol OR "25 hydroxyvitamin D" OR hydroxycholecalciferols OR calcifediol OR calcitriol OR dihydroxycholecalciferols OR ergocalciferols)) AND<br><br>(TITLE-ABS-KEY ("intensive care" OR "newborn intensive care" OR "pediatric intensive care unit" OR "intensive care unit" OR "neonatal intensive care unit" OR "intensive care" OR "critical illness" OR "critical care"))
Web of Science	(TS=("Vitamin D*" OR Cholecalciferol OR "25 hydroxyvitamin D" OR Hydroxycholecalciferols OR Calcifediol OR Calcitriol OR Dihydroxycholecalciferols OR Ergocalciferols)) AND<br><br>TS=("intensive care unit*" OR "paediatric intensive care" OR "neonatal intensive care" OR "critical care" OR "critical illness")

Supplementary 2: Risk of bias assessment for cohort, case-controlled and cross-sectional studies based on Newcastle-Ottawa quality assessment scale

Risk of bias for cohort studies based on Newcastle-Ottawa quality assessment scale (NOS)									
**Study**	**Selection**				**Comparability**	**Outcomes**			**Total**
	Representativeness of the exposed cohort	Selection of the non-exposed cohort	Ascertainment of exposure	Demonstration that outcome of interest was not present at start of study	Comparability of cohorts on the basis of the design or analysis	Assessment of outcome	Was follow-up long enough for outcomes to occur	Adequacy of follow-up of cohorts	
Onwuneme 2015 (37)	1	1	1	1	1	1	1	1	8
Cetinkaya 2017 (46)	1	1	1	0	1	1	1	1	7
Puthuraya 2018 (38)	1	1	1	0	1	1	1	1	7
Kim 2018 (47)	1	1	1	0	1	1	1	1	7
Dogan 2020 (39)	1	1	1	0	1	1	1	1	7

Al-Matary 2021 (40)	1	1	1	1	1	1	1	1	8
Mahmoud 2021 (41)	1	1	1	0	1	1	1	1	7
Matejek 2022 (42)	1	1	1	1	1	1	1	1	8
Jafari 2022 (43)	1	1	1	0	2	1	1	1	9
Saggi 2023 (44)	1	1	1	0	1	1	1	1	7
TurkogluCetin 2024 (45)	1	1	1	0	2	1	1	1	8

Risk of bias for case-controlled studies based on Newcastle-Ottawa quality assessment scale (NOS)

**Study**	**Selection**				**Comparability**	**Exposure**			**Total**
	Case definition adequacy	Representativeness of case	Selection of controls	Definition of controls	Comparability of case and controls on the basis of the design or analysis	Ascertainment of exposure	Same method of ascertainment for cases & controls	Nonresponse rate	

Karatekin 2009 (31)	1	1	0	1	1	1	1	1	7
Prasad 2018 (32)	1	1	0	1	1	1	1	1	7
Dhandai 2018 (33)	1	1	0	1	2	1	1	1	8
Mohamed 2024 (34)	1	1	0	1	1	1	1	1	7
**Risk of bias for cross-sectional studies based on adapted Newcastle-Ottawa quality assessment scale (NOS-xs)**									
**Study**	**Study sample and selection**		**Assessment of exposure and outcome**				**Confounding factors**		**Total**
	Representativeness of the study sample	Sample size	Assessment of the exposure(s)		Assessment of the outcome(s)		Adjustment for confounder(s)	Assessment of confounder(s)	
Mosayebi 2021 (22)	1	1	2		1		1	1	7

Supplementary 3: GRADE risk of bias tool for randomized controlled trials

Study	D1	D2	D3	D4	D5	Overall risk
Ge 2022 (35)	+	+	!	+	+	!
Boskabadi 2024 (36)	+	+	+	+	+	+

+ Low risk
! Some concerns
- High risk

D1 Randomisation process
D2 Deviations from the intended interventions
D3 Missing outcome data
D4 Measurement of the outcome
D5 Selection of the reported result

Supplementary 4:

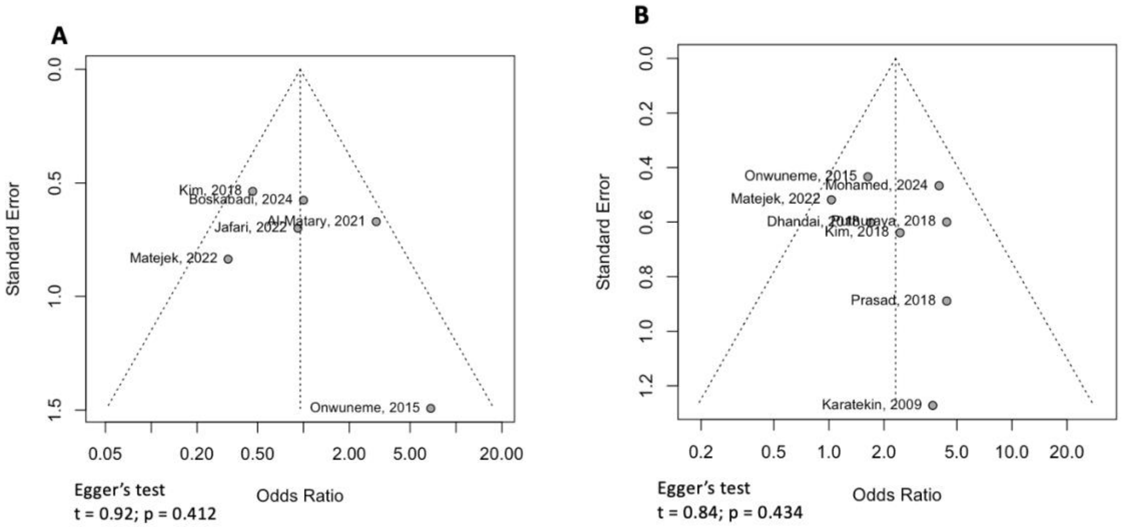

Funnel plots reporting publication bias for (A) All-cause mortality and (B) Incidence of sepsis
